# Interleukin-17 Promotes Migration and Invasion of Human Cancer Cells Through Upregulation of MTA1 Expression

**DOI:** 10.3389/fonc.2019.00546

**Published:** 2019-06-20

**Authors:** Na Guo, Ge Shen, Ying Zhang, Ahmed A. Moustafa, Dongxia Ge, Zongbing You

**Affiliations:** ^1^Department of Structural & Cellular Biology, Tulane University, New Orleans, LA, United States; ^2^Department of Obstetrics and Gynecology, West China Second University Hospital, Sichuan University, Chengdu, China; ^3^Department of Gynecology, Guangyuan First People's Hospital, Guangyuan, China; ^4^Department of Orthopaedic Surgery, Tulane University, New Orleans, LA, United States; ^5^Tulane Cancer Center and Louisiana Cancer Research Consortium, Tulane University, New Orleans, LA, United States; ^6^Tulane Center for Stem Cell Research and Regenerative Medicine, Tulane University, New Orleans, LA, United States; ^7^Tulane Center for Aging, Tulane University, New Orleans, LA, United States; ^8^Southeast Louisiana Veterans Health Care System, New Orleans, LA, United States

**Keywords:** interleukin-17, MTA1, cancer, migration, invasion

## Abstract

Interleukin-17 (IL-17) has been shown to promote development of prostate, colon, skin, lung, breast, and pancreatic cancer. The purpose of this study was to determine if IL-17 regulates MTA1 expression and its biological consequences. Human cervical cancer HeLa and human prostate cancer DU-145 cell lines were used to test if IL-17 regulates metastasis associated 1 (MTA1) mRNA and protein expression using quantitative reverse transcription-polymerase chain reaction and Western blot analysis, respectively. Cell migration and invasion were studied using wound healing assays and invasion chamber assays. Thirty-four human cervical tissues were stained for IL-17 and MTA1 using immunohistochemical staining. We found that IL-17 increased MTA1 mRNA and protein expression in both cell lines. Cell migration was accelerated by IL-17, which was abolished by knockdown of MTA1 expression with small interference RNA (siRNA). Further, cell invasion was enhanced by IL-17, which was eliminated by MTA1 knockdown. Human cervical intra-epithelial neoplasia (CIN) and cervical cancer tissues had increased number of IL-17-positive cells and MTA1 expression compared to normal cervical tissues. The number of IL-17-positive cells was positively correlated with MTA1 expression. These findings demonstrate that IL-17 upregulates MTA1 mRNA and protein expression to promote HeLa and DU-145 cell migration and invasion.

## Introduction

Yao et al. (1, 2) first identified interleukin-17 (IL-17, also named IL-17A) and its first receptor IL-17 receptor A (IL-17RA) (3). IL-17 is produced by T helper 17 (T_H_17) cells, γδ T cells, and other immune cells (4–6). Reddi's lab subcloned IL-17 receptor C (IL-17RC) (7). IL-17RA and IL-17RC form a heterodimer receptor complex for IL-17A and IL-17F (8–11). Steiner et al. (12) reported that IL-17A and IL-17RA expression is increased in prostate cancer and Drake's lab found increased T_H_17 cells in prostate cancer (13). Gupta's lab found IL-17-expressing macrophages and neutrophils in the lesions of proliferative inflammatory atrophy (14)—a precursor to prostatic intraepithelial neoplasia (PIN) and carcinoma (15). Many independent research groups have shown that IL-17 promotes the development of colon (16–19), skin (20, 21), breast (22), lung (23, 24), and pancreatic cancer (25). Our lab has demonstrated that IL-17 promotes development of hormone-naïve prostate cancer and castration-resistant prostate cancer (CRPC) (26, 27).

IL-17 acts alone or synergizes with other stimuli to activate expression of many genes, including cytokines [IL-6, IL-19, IL-20, IL-24, tumor necrosis factor α (TNFα), and granulocyte-colony stimulating factor (G-CSF)], chemokines [IL-8, C-X-C motif ligand 1 (CXCL1), CXCL2, CXCL5, CXCL9, CXCL10, C-C motif ligand 2 (CCL2), CCL7, and CCL20], matrix metalloproteinase (MMP) 13, receptor activator of nuclear factor kappa-B ligand (RANKL), and antimicrobial peptides (lipocalin 2, β-defensin-2, S100A7, and S100A8/9) (28). We previously found that insulin and insulin-like growth factor 1 (IGF1) enhanced IL-17-induced expression of Cxcl1, Ccl20, and Il-6 in mouse embryonic fibroblasts (29). Insulin and IGF1 also acted with IL-17 to increase vascular cell adhesion molecule 1 (VCAM-1) expression in human umbilical vein endothelial cells (HUVECs) (30). We have demonstrated elevated IL-17RC expression in CRPC compared to androgen-dependent prostate cancer and normal prostate (31–33). We found that in human and rodent PIN lesions, IL-17RC levels are elevated to enhance IL-17A-induced activation of nuclear factor- κB (NF-κB) and extracellular signal-regulated kinase ½ (ERK1/2) pathways to increase chemokine/cytokine expression (34). We showed that insulin/IGF1 and IL-17 signaling pathways crosstalk via glycogen synthase kinase 3 (GSK3), which can be blocked by melatonin or pan-Akt inhibitor AZD5363 (29, 35). We revealed that GSK3 phosphorylates IL-17RA at T780 (36). We recently demonstrated that IL-17 induces MMP7 to drive epithelial-to-mesenchymal transition in the prostate (37). Most of these studies have been focused on primary prostate cancer. Few studies have been performed on the role of IL-17 in prostate cancer metastasis. Using an allograft orthotopic mouse prostate cancer model, we found that IL-17 treatment significantly increased metastasis rate compared to the control group (38). Five of the 14 mice with mouse prostate cancer cells co-injected with recombinant IL-17 presented metastases, whereas none of the 13 mice in the control group without IL-17 treatment had any metastases. However, the molecular mechanisms are not clear.

Metastasis-associated gene (MTA) refers to a family of cancer progression-related genes, including MTA1, MTA1s, MTA-ZG29P, MTA2, MTA3, and MTA3L (39). MTA1 is the first gene found in this family, which has been shown to be over-expressed in several human cancers, such as breast, stomach, and colorectal cancer (40). MTA1 gene has 21 exons spreading over a region of about 51-kb in human genome. Alternative splicing from the 21 exons produces 20 transcripts, ranging from 416-base pairs to 2.9-kilobase pairs in length. However, open-reading frames are present only in eight spliced transcripts that code six proteins and two polypeptides and the remaining transcripts are non-coding long RNAs some of which retain intron sequences (41). MTA1 protein interacts with histone deacetylase to form a nucleosome remodeling histone deacetylase (NuRD) complex, which has been shown to regulate oncogenesis (42, 43), angiogenesis (44), and cancer progression of a variety of cancers (45–50). MTA1 is considered as one of the most remarkable indicators associated with cancer progression, aggressive phenotype, and poor prognosis (49). A recent study found that MTA1 silencing in human prostate cancer PC3M cells diminished formation of bone metastases and impaired tumor growth in intracardiac and subcutaneous prostate cancer xenografts, respectively (51). This phenotype was attributed to reduced colony formation, invasion, and migration capabilities of MTA1 knockdown cells (51). MTA1 has been associated with the invasiveness of human prostate cancer cells (52). We previously found that MTA1 expression was decreased in *Il-17rc*-null mouse prostate tumors compared to *Il-17rc*-expressing mouse prostate tumors (27). The purpose of the present study was to determine if IL-17 regulates MTA1 expression and its biological consequences. We found that IL-17 promoted migration and invasion of human cancer cells through upregulation of MTA1 expression.

## Materials and Methods

### Cell Culture

Human prostate cancer cell line DU-145 and human cervical cancer cell line HeLa were obtained from the American Type Culture Collection (Manassas, VA, USA). DU-145 and HeLa cell lines express IL-17RA and IL-17RC (31, 53). The cells were routinely cultured in Dulbecco's Modified Eagle's Medium (DMEM; Caisson Laboratories, Inc., Smithfield, UT) in a humidified 5% CO_2_ incubator at 37°C. The medium contained 10% fetal bovine serum (FBS; Gemini Bio-Products, West Sacramento, CA) without any antibiotics. For induction of MTA1 expression, the cells were cultured in serum-free medium in 60-mm culture dishes and treated without or with 20 ng/ml recombinant human IL-17A (Cat# 7955-IL-025/CF, Fisher Scientific, Pittsburgh, PA) for 8, 16, 24, and 36 h. One group of cells was treated with 10 μg/ml cycloheximide (Fisher Scientific, Pittsburgh, PA) after 24 h of IL-17A treatment and harvested at 36 h. In the siRNA knockdown experiments, the cells were first transfected with 20 nM control siRNA (Cat# 4390844, Silencer® Select Negative Control siRNA, Fisher Scientific, Pittsburgh, PA) or 20 nM siRNA targeting MTA1 (Cat# 4392422, Silencer® Select Pre-designed siRNA, Fisher Scientific, Pittsburgh, PA) using Lipofectamine® 2000 Transfection Reagent (Cat# 11668-019, Invitrogen, Carlsbad, CA) according to the manufacturer's instructions. Twenty-four hours later, the cells were treated without or with 20 ng/ml recombinant human IL-17A for 8, 16, 24, and 36 h.

### Western Blot Analysis

Proteins of the cells were extracted using radioimmunoprecipitation assay (RIPA) lysis buffer and were subjected to 10% sodium dodecyl sulfate -polyacrylamide gel electrophoresis (SDS-PAGE) and transferred to polyvinylidene fluoride (PVDF) membrane using TRANS-BLOT SD Semi-dry Transfer Cell (Bio-Rad Laboratories, Hercules, CA). The membrane was incubated in 5% non-fat dry milk diluted in 1 × Tris-buffered saline with 0.1% Tween 20 (TBS-T) buffer (25 mM Tris-HCl, 125 mM sodium chloride, and 0.1% Tween 20) for 1 h and probed with the indicated primary antibodies overnight. Mouse anti-MTA1 monoclonal antibodies were purchased from Santa Cruz Biotechnology, Inc. (Cat# sc-373765, Dallas, TX; used at 1:100 dilution). Mouse anti-IκBα monoclonal antibodies were obtained from Cell Signaling Technology, Inc. (Cat# 4814S, Danvers, MA; used at 1:1,000 dilution). Rabbit anti-α/β-tubulin monoclonal antibodies were also obtained from Cell Signaling Technology (Cat# 2148; used at 1:1,000 dilution). Mouse anti- glyceraldehyde 3-phosphate dehydrogenase (GAPDH) monoclonal antibodies were purchased from Millipore (Cat# 2955484, Billerica, MA; used at 1:5,000 dilution). After washing 3 times with 1 × TBS-T buffer, the membrane was incubated with IRDye 800CW or IRDye 680RD-conjugated secondary antibodies at 1:5,000 dilution (goat anti-mouse antibodies, Cat# c60107-06 or c40610-09; goat anti-rabbit antibodies, Cat# 41217-03; both from LI-COR Biosciences, Inc., Lincoln, NE) at room temperature for 1 h. The results were visualized and quantified using an Odyssey Infrared Imager (LI-COR Biosciences, Inc., Lincoln, NE). For quantification, MTA1 signals were divided by those of GAPDH to obtain the relative expression of MTA1 protein among the groups.

### Quantitative Real-time Reverse Transcription—Polymerase Chain Reaction (qRT-PCR) Analysis

Total RNA was isolated from the cells treated without or with 20 ng/ml recombinant human IL-17A for 8, 16, 24, and 36 h, using NucleoSpin RNA 250 preps Kit (MACHERY-NAGEL, Inc., Bethlehem, PA). First-strand cDNA synthesis was performed using the PrimeScript^tm^ RT reagent Kit (Cat# RR037A, Takara Bio USA, Inc., Mountain View, CA). Quantitative real-time PCR was performed on QuantStudio 3 instrument (Applied Biosystems, Foster City, CA) using PowerUp™ SYBR Green Master Mix kit (Applied Biosystems, Cat#A25741) following the manufacturer's instructions. The following reaction mixtures were prepared in an optical plate (Cat# TCS0803, Bio-Rad Laboratories, Inc.): 10 μl PowerUp™ SYBR Green Master Mix (2X), 5 μl forward and reverse primers (500 nM for each primer), and 5 μl DNA template (approximately 10 ng) in nuclease-free H_2_O. The plate was sealed with an optical adhesive cover (iCycler iQ® Optical tape, Cat#: 2239444, Bio-Rad Laboratories, Inc.) and then centrifuged briefly to spin down the contents and eliminate any air bubbles. The conditions for quantitative PCR reactions were set up on QuantStudio 3 as following: one cycle of 50°C for 2 min, one cycle of 95°C for 2 min, and 40 cycles of 95°C for 15 s and 60°C for 1 min. At the end of the PCR reactions, the samples were subjected to a melting analysis to confirm specificity of the amplicons. The PCR primer sequences were: MTA1 forward: 5′-GCAGCTGAAGCTGAGAGCAAGTTA-3′; MTA1 reverse: 5′-CCTTGACGTTGTTGACGCTGA-3′; GAPDH forward: 5′-CCACTCCTCCACCTTTGAC-3′; GAPDH reverse: 5′-ACCCTGTTGCTGTAGCCA-3′. The results were normalized by GAPDH levels using the formula ΔCt (Cycle threshold) = Ct of target gene**–**Ct of GAPDH. The fold change of mRNA level of each treatment group was calculated as: ΔΔCt = ΔCt of target gene in the treatment group–ΔCt of target gene in the control group, and fold change = 2^−ΔΔCt^.

### Wound Healing Assay

HeLa and DU-145 cells were cultured in DMEM supplemented with 10% FBS in 60-mm tissue culture dishes until they reached 100% confluence as a monolayer. The monolayer was scratched with a sterile 1,000-μl pipette tip across the center of each dish. After scratching, the dishes were gently washed twice with DMEM to remove the detached cells. The dishes were replenished with serum-free medium. The control group was treated with 0.1% bovine serum albumin (BSA) in phosphate-buffered saline (PBS), while 20 ng/ml recombinant human IL-17A (dissolved in 0.1% BSA in PBS) was added into the medium of the treatment group. In siRNA knockdown experiments, the cells were first transfected with 20 nM control siRNA or 20 nM siRNA targeting MTA1 using Lipofectamine® 2000 Transfection Reagent as described above; 24 h later, the monolayer cells were scratched and treated with 20 ng/ml recombinant human IL-17A. Photomicrographs of the wounds were taken from time zero and then every 24 h under an EVOS FL Auto Microscope (Life Technologies Inc., Carlsbad, CA). Using ImageJ software, horizontal gap distances at 5 points along each wound were measured and averaged to represent the width of the wound. Wound healing rate was calculated as (wound width of time zero–that of each time point) ÷ wound width of time zero × 100%.

### Invasion Assay

Invasion assay was performed using Corning® BioCoat™ Matrigel® Invasion Chambers (Corning Inc., Corning, NY) following the manufacturer's instructions. The cells were first transfected with 20 nM control siRNA or 20 nM siRNA targeting MTA1 using Lipofectamine® 2000 Transfection Reagent according to the manufacturer's instructions; 24 h later, approximately 2 × 10^5^ cells were seeded in each upper chamber in serum-free medium without or with 20 ng/ml recombinant human IL-17A in triplicate wells per group, while the lower chamber contained medium with 10% FBS. After 24 h, non-invading cells were removed from the upper chamber with a cotton swab; the cells invaded through the Matrigel®-coated porous membrane were fixed with methanol, stained with 0.5% crystal violet, and counted under a microscope. For HeLa cells, one picture per well under 100 × magnification was taken, which covered almost the entire porous membrane. For DU-145 cells, pictures of 5 regions of interest (1 center and 4 corners) were taken under 200 × magnification, because there were too many cells to count on the whole membrane.

### Immunohistochemical (IHC) Staining of Human Cervical Tissues

The study was conducted in accordance with the Declaration of Helsinki (revised in 2013). The use of archived and de-identified human tissue specimens was approved by the Institutional Review Board of Tulane University (Protocol #394164, approved on November 25, 2015). Thirty-four human cervical tissue specimens, including 5 cases of normal cervix, 5 cases of cervical intra-epithelial neoplasia (CIN) I, 5 cases of CIN II, 5 cases of CIN III, and 14 cases of cervical squamous carcinoma were previously collected during 2017 to 2018 and archived at Guangyuan First People's Hospital, Guangyuan, Sichuan Province, China. The 5 normal cervical tissues were obtained from patients (age 48–52 years) undertaking hysterectomy due to uterine myomas; 15 CIN specimens were obtained from patients (age 34–49 years) who received cervical biopsies or conization therapies; and 14 cervical cancer specimens were obtained from patients (age 44–55 years) who received radical hysterectomy due to cervical squamous carcinoma (stage Ib-IIa). Inclusion criteria were: (1) patients with clear pathological diagnosis of the cervical lesions; and (2) patients had signed informed consent prior to the surgical procedures. Exclusion criteria were: (1) patients with concurrent autoimmune disease, active or chronic infection, cardiovascular disease, or connective tissue disease; or (2) patients with a history of other malignant tumors; or (3) patients who received immunosuppressive treatment, radiotherapy, or chemotherapy prior to the surgical procedures. The specimens were fixed with 10% formalin, embedded in paraffin blocks, and cut into 4-μm thick tissue sections. All specimens had been de-identified prior to being provided to the investigators. IHC staining followed our previously published protocol (26, 36, 37). The primary antibodies used were: goat anti-human IL-17/IL-17A polyclonal antibodies (1:40 dilution, Cat# AF-317-NA, R&D systems, Inc., Minneapolis, MN) and mouse anti-MTA1 monoclonal antibodies (1:25 dilution, Cat# sc-373765, Santa Cruz Biotechnology, Inc., Dallas, TX). VECTASTAIN ABC Kits and DAB Peroxidase Substrate Kit (Vector laboratories, Inc., Burlingame, CA) were used according to the manufacturer's instructions. For IL-17 staining, 5 regions were randomly selected and the number of IL-17-positive cells were counted in 5 high-power (400x) fields under a microscope. For MTA1 staining, a total score (proportion score + intensity score, range 0–8) was graded according to the Allred scoring system (32, 54).

### Statistical Analyses

Quantitative data are presented as the mean ± standard deviation and compared using one-way analysis of variance (ANOVA) followed by Student's *t*-test. Correlation of IL-17-positive cell number and MTA1 staining score was analyzed with Pearson correlation analysis. Differences between the groups were considered statistically significant when *p* < 0.05.

## Results

### IL-17 Induces Expression of MTA1 at mRNA and Protein Levels

**Figure 1:**
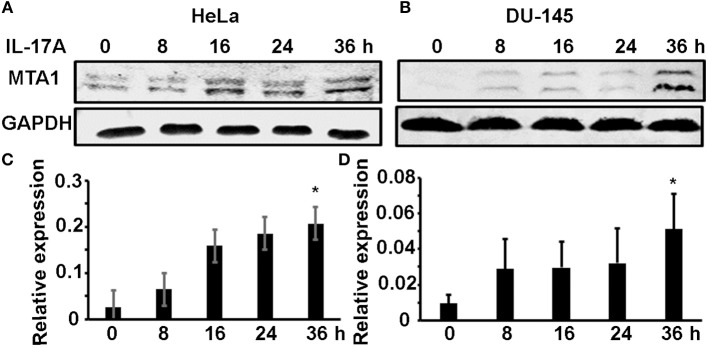
IL-17A induces MTA1 protein expression in human cancer cells. HeLa and DU-145 cells were treated with 20 ng/ml recombinant human IL-17A for the indicated time points. **(A,B)** Representative Western blot analysis of MTA1 protein expression, using GAPDH levels as loading controls. **(C,D)** Relative expression of MTA1 normalized by GAPDH levels; data represent mean ± standard deviation (SD, error bars) of 3 independent experiments; ^*^p < 0.05 between the IL-17A-treated group and the time zero (untreated control) group.

We previously found that MTA1 expression was decreased in *Il-17rc*-null mouse prostate tumors compared to *Il-17rc*-expressing mouse prostate tumors (27). Therefore, we hypothesized that MTA1 might be an IL-17-downstream target gene. To test this hypothesis, we used two human cancer cell lines HeLa (human cervical cancer cell line) and DU-145 (human prostate cancer cell line). HeLa and DU-145 cells were treated with 20 ng/ml recombinant human IL-17A for 8, 16, 24, and 36 h. MTA1 protein levels were assessed with Western blot, using GAPDH as loading control. We found that IL-17A induced MTA1 protein expression starting at 8 h through 36 h in HeLa cells (Figure 1A). Similarly, we found that IL-17A induced MTA1 protein expression in DU-145 cells, though at lower amplitudes from 8 to 24 h (Figure 1B). After normalization based on GAPDH levels, we found that IL-17A significantly induced MTA1 protein expression at 36 h in both HeLa and DU-145 cells (Figures 1C,D).

**Figure 2:**
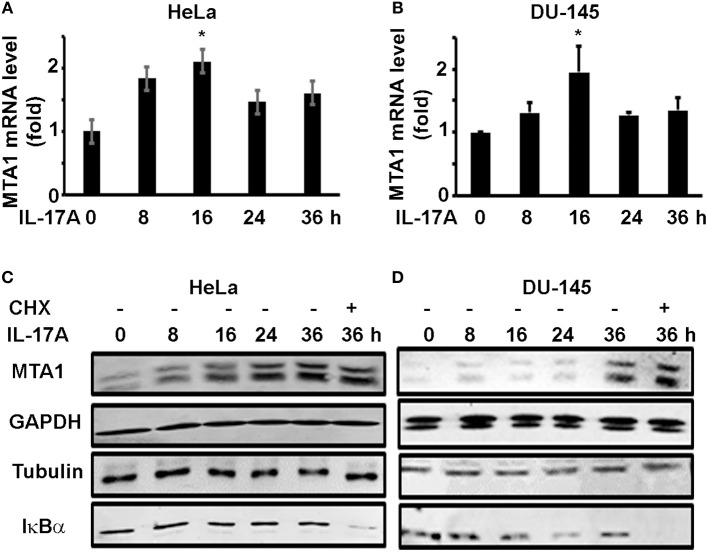
IL-17A induces MTA1 mRNA expression in human cancer cells. HeLa and DU-145 cells were treated with 20 ng/ml recombinant human IL-17A for the indicated time points. **(A,B)** qRT-PCR analysis of MTA1 mRNA expression; data represent mean ± standard deviation (SD, error bars) of 3 independent experiments; ^*^p < 0.05 between the IL-17A-treated group and the time zero (untreated control) group. **(C,D)** Representative Western blot analysis of protein expression; the cells were treated with 20 ng/ml recombinant human IL-17A for 8–36 h; one group was treated with 10 μg/ml cycloheximide (CHX) at 24 h and harvested at 36 h.

To check if IL-17A induces MTA1 mRNA expression, we similarly treated HeLa and DU-145 cells and performed quantitative real-time reverse transcription—polymerase chain reaction (qRT-PCR) analysis of MTA1 mRNA expression. We found that IL-17A induced MTA1 mRNA expression in both HeLa and DU-145 cells (Figures 2A,B). However, we observed that the peak levels of MTA1 mRNA expression were at 16 h after IL-17A treatment, whereas the peak levels of MTA1 protein expression were at 36 h (Figures 1A,B). We speculated that this difference might be caused by the fact that MTA1 protein is relatively stable. To confirm our speculation, we added a group of cells that were treated with 10 μg/ml cycloheximide (CHX, an inhibitor of protein translation) at 24 h after IL-17A treatment. We found that CHX-treated group showed MTA1 protein levels similar to the CHX-untreated group at 36 h in both HeLa and DU-145 cells (Figures 2C,D). Similar findings were found with GAPDH and tubulin proteins, two well-known stable proteins. Yet, 10 μg/ml CHX was able to reduce the levels of IκBα protein, a well-known short-lived protein (Figures 2C,D), suggesting that the CHX dosage was effective.

### IL-17 Promotes Migration of Human Cancer Cells

**Figure 3:**
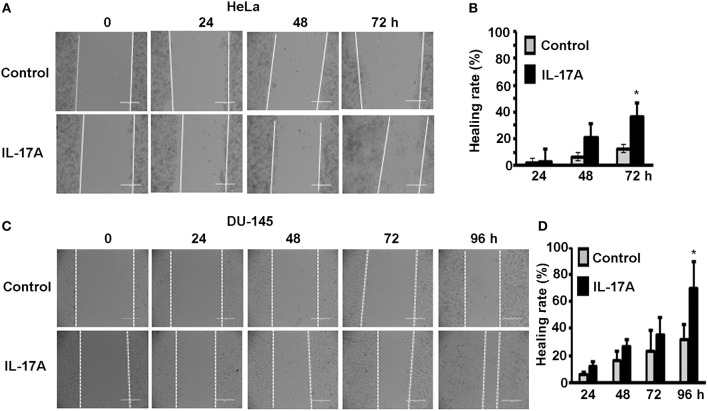
IL-17A promotes migration of human cancer cells. Confluent monolayer of HeLa and DU-145 cells was scratched to make a wound and then treated without (control group) or with 20 ng/ml recombinant human IL-17A for the indicated time points. **(A,C)** Representative photomicrographs of the wounds; dotted lines mark the front edges of migrating cells; scale bar, 400 μm. **(B,D)** Wound healing rate was calculated as (wound width of time zero–that of each time point) ÷ wound width of time zero × 100%; data represent mean ± standard deviation (SD, error bars) of 3 independent experiments; ^*^p < 0.05 between the IL-17A-treated group and the untreated control group.

Since MTA1 has originally been found to be associated with cancer metastasis, we investigated if IL-17 could promote cancer cell migration using wound healing assays. HeLa and DU-145 cells were grown to complete confluence in 60-mm culture dishes and a wound was made by scratching the monolayer cells with a sterile pipette tip. The cells were either untreated (control group) or treated with 20 ng/ml recombinant human IL-17A. Photomicrographs were taken every 24 h up to 96 h. We found that IL-17A treatment significantly accelerated the wound healing of HeLa monolayer cells at 72 h, compared to the control group (Figures 3A,B). Similarly, we found that IL-17A treatment significantly accelerated the wound healing of DU-145 monolayer cells at 96 h, compared to the control group (Figures 3C,D).

**Figure 4:**
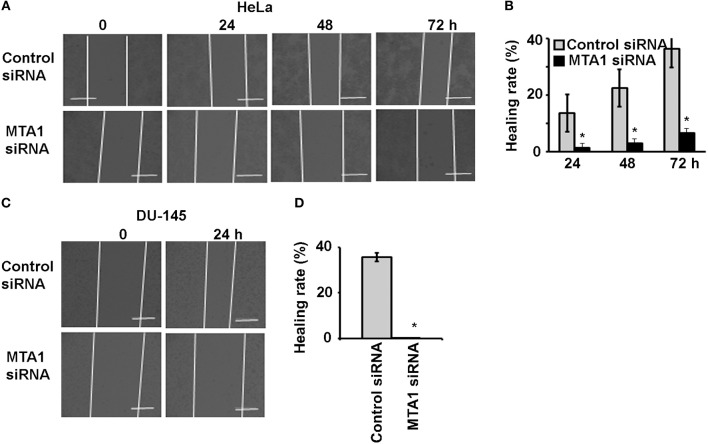
IL-17A acts through MTA1 to promote migration of human cancer cells. HeLa and DU-145 cells were transfected with either control siRNA or siRNA targeting MTA1. Confluent monolayer of both control siRNA and MTA1 siRNA groups was scratched to make a wound and then treated with 20 ng/ml recombinant human IL-17A for the indicated time points. **(A,C)** Representative photomicrographs of the wounds; dotted lines mark the front edges of migrating cells; scale bar, 400 μm. **(B,D)** Wound healing rate was calculated as (wound width of time zero–that of each time point) ÷ wound width of time zero × 100%; data represent mean ± standard deviation (SD, error bars) of 3 independent experiments; ^*^p < 0.05 between the MTA1 siRNA group and the control siRNA group.

To check if MTA1 plays any role in cancer cell migration, we transfected the cells with either siRNA targeting MTA1 or negative control siRNA and performed wound healing assays. We found that MTA1 siRNA-transfection significantly inhibited IL-17A-induced migration of HeLa cells (Figures 4A,B) and DU-145 cells (Figures 4C,D).

### IL-17 Promotes Invasion of Human Cancer Cells

**Figure 5:**
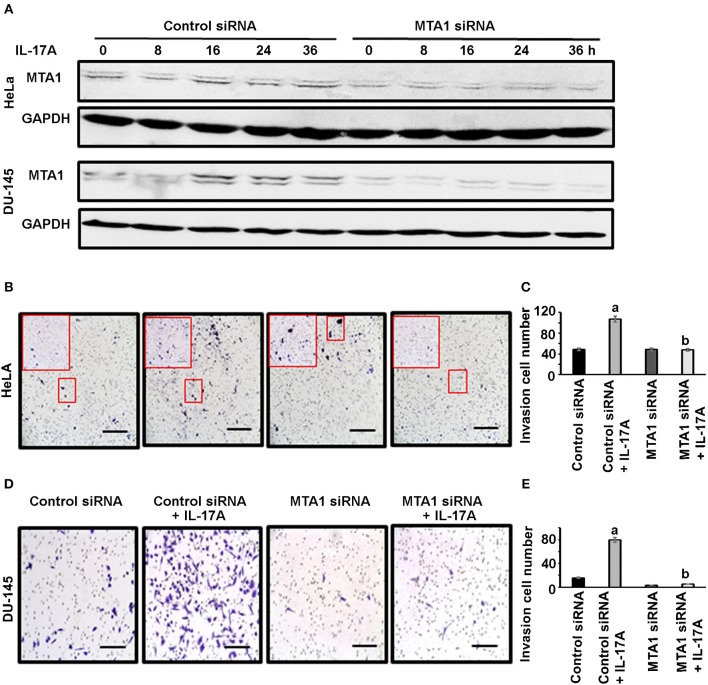
IL-17A acts through MTA1 to promote invasion of human cancer cells. HeLa and DU-145 cells were transfected with either control siRNA or siRNA targeting MTA1. Invasion assays were performed using Matrigel®-coated invasion chambers. Approximately 2 x10^5^ cells were seeded in each upper chamber in serum-free medium without or with 20 ng/ml recombinant human IL-17A in triplicate wells per group, while the lower chamber contained medium with 10% FBS. After 24 h, non-invading cells were removed from the upper chamber; the cells invaded through the Matrigel®-coated porous membrane were fixed with methanol, stained with 0.5% crystal violet, and counted under a microscope. **(A)** Western blot analyses of MTA1 expression in HeLa and DU-145 cells transfected with control siRNA and MTA1 siRNA, with 20 ng/ml recombinant human IL-17A treatment for the indicated time. **(B,D)** Representative photomicrographs of the porous membrane with stained cells; for HeLa cells, one picture per well under 100 × magnification (scale bar, 400 μm) was taken, which covered almost the entire porous membrane, and the inserted frame showed the stained cells under 200 × magnification; for DU-145 cells, one representative of 5 regions taken under 200 × magnification (scale bar, 200 μm) was shown. **(C,E)** Number of the cells invaded through the porous membrane; data represent mean ± standard deviation (SD, error bars) of 3 independent experiments; a, *p* < 0.01 between the control siRNA + IL-17A group and the control siRNA group; b, *p* < 0.01 between the MTA1 siRNA + IL-17A group and the control siRNA + IL-17A group; there was no significant difference between the MTA1 siRNA + IL-17A group and the MTA1 siRNA group (*p* > 0.05).

Next, we checked if MTA1 plays any role in cancer cell invasion using Corning® BioCoat™ Matrigel® Invasion Chambers. HeLa and DU-145 cells were first transfected with either siRNA targeting MTA1 or negative control siRNA. To confirm that MTA1 expression was knocked down with the siRNA, we performed Western blot analysis. We found that IL-17A induced MTA1 expression in both HeLa and DU-145 cells transfected with the control siRNA, however, transfection with MTA1 siRNA reduced both the basal levels of and IL-17A-induced MTA1 expression (Figure 5A). Then, the cells were plated in the upper chamber in serum-free medium with or without 20 ng/ml recombinant human IL-17A, while the lower chamber contained medium with 10% fetal bovine serum (FBS). Twenty-four hours later, the cells invaded through the Matrigel®-coated porous membrane were stained with 0.5% crystal violet and counted. We found that IL-17A increased invasion of HeLa cells transfected with control siRNA, whereas IL-17A failed to increase invasion of HeLa cells transfected with MTA1 siRNA (Figures 5B,C). Similar findings were obtained in DU-145 cells (Figures 5D,E).

### IL-17A-Positive Cell Number Correlates With MTA1 Expression in Human Cervical Tissues

**Figure 6:**
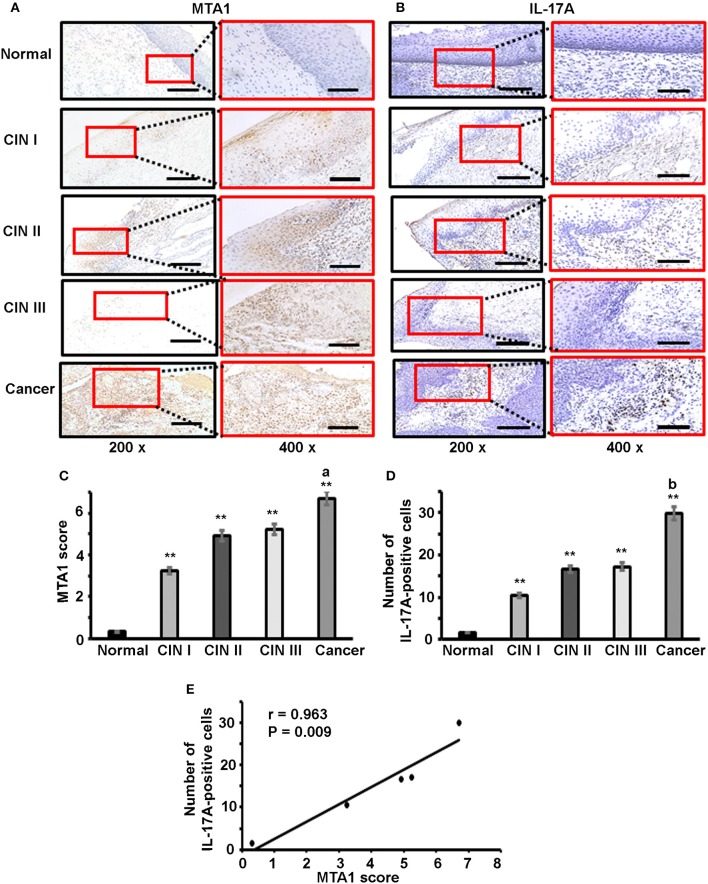
Immunohistochemical staining of IL-17 and MTA1 protein in human cervical tissues. **(A)** Representative photomicrographs of MTA1 staining; scale bar, 200 μm for 200 × magnification and 100 μm for 400 × magnification. **(B)** Representative photomicrographs of IL-17 staining; scale bar, 200 μm for 200 × magnification and 100 μm for 400 × magnification. **(C)** Quantification of MTA1 staining using the Allred scoring system; data represent mean ± standard deviation (SD, error bars); ^**^p < 0.01 between CIN/cancer group and normal cervix group; a, *p* < 0.05 between cervical cancer group and CIN III group; **(D)** Quantification of IL-17 staining by counting the number of IL-17-positive cells; data represent mean ± standard deviation (SD, error bars); ^**^p < 0.01 between CIN/cancer group and normal cervix group; b, *p* < 0.05 between cervical cancer group and CIN III group; **(E)** Correlation analysis of the number of IL-17-positive cells and MTA1 score; the number of IL-17-positive cells was counted in the areas of 5 high-power (400x) fields and the average number represents each sample.

To check if our *in vitro* findings might have any relevance in human diseases, we examined IL-17A and MTA1 expression in human cervical tissue specimens, including 5 cases of normal cervix, 5 cases of cervical intra-epithelial neoplasia (CIN) I, 5 cases of CIN II, 5 cases of CIN III, and 14 cases of cervical squamous carcinoma. Immunohistochemical staining (IHC) was performed. We found little MTA1 staining and few IL-17A-positive cells in normal cervical tissues, however, MTA1 staining and number of IL-17A-positive cells were increased in CIN and cervical cancer tissues (Figures 6A,B). MTA1 staining was quantified using the Allred scoring system (54). We found that MTA1 staining was significantly increased in CIN and cancer tissues compared to normal cervical tissues (Figure 6C). There was no significant difference among CIN I to III, but MTA1 staining in cervical cancer was significantly increased compared to CIN I to III (Figure 6C). Likewise, we found that the number of IL-17A-positive cells was significantly increased in CIN and cancer tissues compared to normal cervical tissues (Figure 6D). There was no significant difference among CIN I to III, but the number of IL-17A-positive cells in cervical cancer was significantly increased compared to CIN I to III (Figure 6D). Given the similarity of the patterns of expression between IL-17A and MTA1, we did correlation analysis and found that the number of IL-17A-positive cells was positively correlated with MTA1 staining (Figure 6E).

## Discussion

In the present study, we found that IL-17 induced mRNA and protein expression of MTA1 in human HeLa and DU-145 cancer cells. To the best of our knowledge, this is the first time that MTA1 has been found to be an IL-17 target gene. We also found that MTA1 was responsible for IL-17-induced migration and invasion of HeLa and DU-145 cells, as MTA1 knockdown abolished IL-17-induced migration and invasion of the cancer cells. Our findings are in consistence with the previous report in regard to MTA1's role in cell migration and invasion. Of note, our wound healing assays were performed in a time-frame of 24–96 h, thus it is reasonable to suspect that cell proliferation might be a factor in the wound closure. However, we and others have shown that IL-17 does not directly affect cell proliferation *in vitro* (34, 55), thus the rates of cell proliferation in the control group and IL-17-treated group are comparable. Other studies have shown that MTA1 expression is correlated with prostate cancer progression (56), angiogenesis (52), and metastasis (57). Given that we have previously shown that IL-17 promotes development of prostate cancer (26), we reason that IL-17 may at least partially act through upregulating MTA1 to promote prostate cancer formation based on the findings from the present study.

A previous study has found that IL-17 was expressed by neutrophils, mast cells, innate lymphoid cells, and T_H_17 cells in human cervical cancer (58). Another study found that IL-17 levels in cervical tissue homogenates of CIN and cervical cancer were significantly higher than that in normal cervical tissue homogenate (59). MTA1 expression level has been associated with migration and invasion of cervical cancer cells (60). In the present study, we found that IL-17-induced migration and invasion of HeLa cells was dependent on MTA1 expression as MTA1 knockdown abolished IL-17-induced migration and invasion. In the human cervical specimens, we found that the number of IL-17-positive cells was positively correlated with MTA1 expression, suggesting that increased IL-17 expression in CIN and cervical cancer tissues may upregulate MTA1 expression, which facilitates neoplastic cell migration and invasion. A limitation of the present study is that the identity of the IL-17-positive cells requires further experiments. Double staining of IL-17 and MTA-1 was not performed because it is believed that IL-17 was expressed by immune cells whereas MTA-1 was expressed by epithelial cells. Another limitation of the present study is that other MTA family members are not tested, thus it is unknown if IL-17's effect is unique to MTA1, which shall be examined in future studies.

In conclusion, the present study shows that IL-17 upregulates MTA1 mRNA and protein expression to promote HeLa and DU-145 cell migration and invasion. This new function of IL-17 may play a role in development of invasive cervical cancer and prostate cancer. Future studies shall explore whether IL-17 promotes cancer metastasis through upregulation of MTA1 expression.

## Data Availability

All datasets generated for this study are included in the manuscript and/or the supplementary files.

## Author Contributions

NG and GS performed the experiments, analyzed the data, and prepared the manuscript draft. YZ provided the human specimens. AM and DG set up the experiments and repeated the key experiments. ZY conceived the work, analyzed the data, and prepared the manuscript. All authors critically revised the manuscript, approved the final version, and agreed to be accountable for all aspects of the manuscript.

### Conflict of Interest Statement

The authors declare that the research was conducted in the absence of any commercial or financial relationships that could be construed as a potential conflict of interest.

## Abbreviations

CIN: cervical intra-epithelial neoplasia
Ct: cycle threshold
DMEM: Dulbecco's Modified Eagle's Medium
GAPDH: glyceraldehyde 3-phosphate dehydrogenase
IHC: immunohistochemical staining
IL-17: Interleukin-17
IL-17A: Interleukin-17A
IL-17RC: Interleukin-17 receptor C
MTA1: metastasis associated 1
siRNA: small interference RNA.
